# Ultrafast Pulse Generation from Quantum Cascade Lasers

**DOI:** 10.3390/mi13122063

**Published:** 2022-11-24

**Authors:** Feihu Wang, Xiaoqiong Qi, Zhichao Chen, Manijeh Razeghi, Sukhdeep Dhillon

**Affiliations:** 1 Shenzhen Institute for Quantum Science and Engineering, Southern University of Science and Technology, Shenzhen 518055, China; 2 International Quantum Academy, Shenzhen 518048, China; 3 Guangdong Provincial Key Laboratory of Quantum Science and Engineering, Southern University of Science and Technology, Shenzhen 518055, China; 4 School of Information Technology and Electrical Engineering, The University of Queensland, Brisbane, QLD 4072, Australia; 5 Center for Quantum Devices, Department of Electrical Engineering and Computer Science, Northwestern University, Evanston, IL 60208, USA; 6 Laboratoire de Physique de l’Ecole Normale Supérieure, ENS, Université PSL, CNRS, Sorbonne Université, Université de Paris, 75014 Paris, France

**Keywords:** quantum cascade lasers, mode-locking, ultrafast dynamics, terahertz and mid-infrared, semiconductor lasers, pulse compression, laser physics

## Abstract

Quantum cascade lasers (QCLs) have broken the spectral barriers of semiconductor lasers and enabled a range of applications in the mid-infrared (MIR) and terahertz (THz) regimes. However, until recently, generating ultrashort and intense pulses from QCLs has been difficult. This would be useful to study ultrafast processes in MIR and THz using the targeted wavelength-by-design properties of QCLs. Since the first demonstration in 2009, mode-locking of QCLs has undergone considerable development in the past decade, which includes revealing the underlying mechanism of pulse formation, the development of an ultrafast THz detection technique, and the invention of novel pulse compression technology, etc. Here, we review the history and recent progress of ultrafast pulse generation from QCLs in both the THz and MIR regimes.

## 1. Introduction

Quantum cascade lasers (QCLs) are electrically pumped compact semiconductor light sources that were first demonstrated in the mid-infrared in 1994 by Faist et al. at Bell Lab [1] and in the terahertz (THz) frequency range by Köhler et al. at Scuola Normale Superiore in 2002 [2]. The QCL concept has enabled powerful and compact coherent light sources in previously inaccessible or unpractical mid-infrared and THz regions of the electromagnetic spectrum. In the mid-infrared area, QCLs have achieved an impressive performance with more than 5.6 W output power from a single facet [3,4,5,6], and with high wall-plug efficiency up to 31% at room temperature (RT) in a continuous wave (CW) operation [7]. Besides, high beam-quality single-mode long-wave infrared (LWIR) QCLs with record light extraction (2.0 MW cm^−2^ sr^−1^ for λ ≈ 10 μm, 2.2 MW cm^−2^ sr^−1^ for λ ≈ 9 μm, 5.0 MW cm^−2^ sr^−1^ for λ ≈ 8 μm) from a single facet in CW operation at 15 °C have also been demonstrated [8]. These results mark an important milestone in the lighting capability of inter-sub-band semiconductor lasers in the mid-infrared spectral ranges. Beyond the Restrahlen band (>50 µm), QCLs have also shown remarkable development: high output power over 1 W, far-field engineering on metal–metal waveguide, quantum limited linewidths and self-generated frequency combs have been demonstrated [9,10,11,12,13]. Although there remain challenges, the further development and exploitation of QCLs is crucial due to the unparalleled success of these devices in terms of their output power and wavelength agility in a compact, potentially inexpensive and user-friendly geometry.

Mode-locking of QCLs enables the gathering of mid-infrared and THz energy on a very short timescale and generation of periodic light pulses in a long-term time domain. It offers unique conditions and an extreme environment for the development of cutting-edge technology, the test of fundamental physics, the examination of relativity theory, and expansion of the boundaries of human cognition, etc. [14,15,16,17,18]. Unlike traditional semiconductor lasers, mode-locking of QCLs has proven to be extremely challenging due to its ultrafast gain recovery time, which is more than one order of magnitude smaller than the round-trip time of photons that are circling in the laser cavity [19,20,21]. However, this stumbling block was overcome in 2011 by injecting a round-trip electrical modulation to force pulse formation within the laser cavity [22]. Thereafter, ultrafast QCLs have undergone considerable development, including the explanation of the underlying physics of mode-locking [23,24,25], pulse shortening by a novel dispersion compensation technique [26], and other pulse-generation and compression techniques [24,27,28].

Here, we focus on discussing the development of ultrafast pulse generation from QCL, and divide the main content of this review paper into four sections, as follows: (I) in the first section, we give an introduction to the ultrafast dynamics of QCLs from the theoretical aspect [28,29,30,31]; (II) in the second section, we present the high-speed modulation of QCLs using a radio-frequency (RF) injection locking; (III) in the third section we present the mode-locked QCLs [22,32,33,34,35]; (IV) in the fourth section, we present the state-of-art results of ultrafast pulse generation from THz QCLs [26], and (V) in the fifth section we present pulse generation in MIR-QCLs by applying novel compression techniques [24,27].

## 2. Ultrafast Dynamics of QCLs

As unipolar devices, photon emission in QCLs is based on intersubband transitions in the conduction band of quantum heterostructures. They exhibit ultra-short carrier lifetimes that are on the same (picosecond) scale as the photon lifetime, which leads to the absence of the relaxation resonant oscillations in the transient response of these devices and ultrafast gain dynamics. The ultrafast gain dynamics of QCLs, combined with Kerr nonlinearities, the group velocity dispersions (material dispersion, waveguide dispersion, and gain dispersion), and spatial hole burning determine the pulse formation dynamics in QCLs. These intersubband transitions feature strong third-order optical nonlinearities, due to the large optical matrix element between the excited states and the empty lower states, allowing parametric processes due to four-wave mixing (FWM) [36]. Through the cascade FWM process based on multiple laser longitudinal modes and low group velocity dispersion (GVD), free-running combs with frequency modulations have been achieved in MIR-QCLs (this is frequency modulated QCLs and hence in principle no pulse generation) [36,37]. In addition, it has been found that a finite linewidth enhancement factor in fast gain medium lasers leads to a considerable Kerr nonlinearities, more so than in interband lasers with slow gain dynamics, which means that shorter carrier lifetimes lead to a wider FWM gain bandwidth, which in turn supports wider multi-mode emission [37]. Carrier lifetimes in THz QCLs are an order of magnitude higher than that in MIR-QCLs, which in principle make pulse formation in THz QCLs easier than in MIR-QCLs. However, the semiconductor material is more dispersive at THz frequencies than in mid-infrared frequencies due to stronger coupling with the crystalline lattice (for instance, GVD of GaAs at 40 K at 3.5 THz is 250 times higher that at 7 µm) [12], thus dispersion compensation techniques, such as a chirped corrugation etched into the facet of the laser [12] or GTIs [26], have to be considered to form stable pulses. In multi-mode QCLs with Fabry–Perot cavities, the waves travelling in forward and backward directions are coupled as they share the same gain medium, which gives rise to spatial hole burning (SHB) that favors multi-mode emission and can help to further reduce the pulse duration of the short pulses in QCLs. However, on the other hand, SHB also results in pulse instabilities and non-stationary pulse generation [38]. Furthermore, it has been concluded that the combined effects of SHB, GVD and Kerr nonlinearities due to asymmetric gain give rise to the recently observed linear frequency chirp [39]. The self-starting frequency combs generated in QCLs can be improved by RF injection through active mode-locking [22,23] and even with harmonic mode-locking [28], which provides possibilities for higher repetition rates beyond the limitation from the laser cavity length. Recently, soliton structures have been observed in ring QCLs, which opens interesting physics questions in the lasers with fast gain dynamics.

The theoretical models used to investigate multi-mode dynamics and QCL combs include reduced rate equations [40,41], Maxwell Bloch equations [25,42], and Master equations [29,39,43,44]. The multi-mode reduced rate equations (Equations (1)–(4)) are based on interactions between electrons and photons through stimulated emissions, spontaneous emission, and stimulated absorptions. This model is very suitable for studying the time-resolved electron and photon transport dynamics and the steady-state analysis of the laser, such as light-current-voltage (LIV) curves. This model has been used to study the frequency tuning mechanisms [40] and ultra-fast mode switching dynamics in coupled-cavity QCLs [29]. It is also adapted to include the external perturbations into the model, such as optical injections and optical feedback effects. The model has been used to study single-mode and multi-mode dynamics under optical feedback in QCLs [41]. However, as rate equations, this model does not include spatial dependence effects, such as SHB.

(1) $$\frac{dN_{3}\left(t\right)}{dt}=\frac{\eta_{3}I}{q}-\sum_{m}G_{m}\left(N_{3}\left(t\right)-N_{2}\left(t\right)\right)S_{m}\left(t\right)-\frac{N_{3}\left(t\right)}{\tau_{3}},$$

(2) $$\frac{dN_{2}\left(t\right)}{dt}=\frac{\eta_{2}I}{q}+\underset{m}{\sum }G_{m}\left(N_{3}\left(t\right)-N_{2}\left(t\right)\right)S_{m}\left(t\right)+\frac{N_{3}\left(t\right)}{\tau_{32}}+\frac{N_{3}\left(t\right)}{\tau_{\mathrm{sp}}}-\frac{N_{2}\left(t\right)}{\tau_{2}},$$

(3) $$\frac{dS_{m}\left(t\right)}{dt}=MG_{m}\left(N_{3}\left(t\right)-N_{2}\left(t\right)\right)S_{m}\left(t\right)+\frac{M\beta_{\mathrm{sp}}N_{3}\left(t\right)}{\tau_{\mathrm{sp}}}-\frac{S_{m}\left(t\right)}{\tau_{p,m}},$$

(4) $$\frac{d\varphi_{m}\left(t\right)}{dt}=\frac{\alpha }{2}\left(MG_{m}\left(N_{3}\left(t\right)-N_{2}\left(t\right)\right)-\frac{1}{\tau_{p,m}}\right),$$

where $N_{3}\left(t\right)$ and $N_{2}\left(t\right)$ are the carrier populations in the upper and lower laser levels of the active medium (ULL and LLL), respectively, and $S_{m}\left(t\right)$ and $\varphi_{m}\left(t\right)$ are the photon population and the phase of the electric field in longitudinal mode *m*. The other input parameters include the injection efficiencies into ULL and LLL $\eta_{3}$ and $\eta_{2}$, the drive current *I*, the number of periods in the active cavity *M*, spontaneous emission factor $\beta_{\mathrm{sp}}$, the carrier lifetimes $\tau_{3}$, $\tau_{32}$, $\tau_{2}$ and photon lifetime $\tau_{p}$, the spontaneous emission lifetime $\tau_{\mathrm{sp}}$, the linewidth enhancement factor α and the gain factor for mode *m* $G_{m}$. The gain recovery time in QCLs can be described by the total carrier lifetime in ULL $\tau_{3}$ in this model. The dependence of the optical gain on the population inversion and the amplitude-to-phase coupling are also included in this model.

The Maxwell–Bloch equations combine the Bloch equation and the wave equation, and are a set of equations for the normalized envelope of the electric field, the polarization, and the population inversion in the gain medium. By considering the polarization of the electric field, which describes the interactions between the laser field and the gain medium, this model includes the effects of Kerr nonlinearities through the optical susceptibility. It also includes the coherent coupling between the populations, such as Risken–Nummedal–Graham–Haken (RNGH) instabilities induced by the coherent resonant tunneling between adjacent stages in the active region. In addition, this model has time and spatial (only z direction) as independent parameters, which can include the SHB effects originated from the standing waves in the FP laser cavities, which play an important role on multimode operation and pulse duration reduction in the QCL combs study. This model has been used to investigate self-starting mode-locking and the formation of optical instabilities in QCLs [45,46]. However, as a full model, it is difficult to understand the roles of each of the physical effects on the formed frequency combs or pulses in the QCLs.

**Figure 1:**
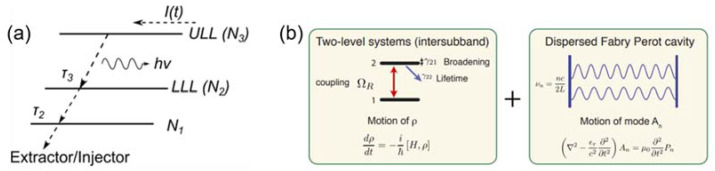
The theoretical models used to study multi-mode dynamics and frequency combs in QCLs: (**a**) the electron transportations in QCLs based on which the reduced rate equation model was developed; (**b**) Maxwell–Bloch Equations that combine the Bloch equation and the wave equation to study optical frequency combs in QCLs. (Figure modified from Ref [47], reprint with the permission of Optica publishing group).

The conventional Haus Master equation can be used to study how the pulse shape varies under the gain dispersion and Kerr nonlinearities in conventional diode lasers where the gain dynamics are not fast, such that the gain recovery time is longer than one laser round trip time as shown in Figure 1 [47]. Despite its popularity, the Haus Master equation approach does not account for light-matter coherent effects and, additionally in the case of active mode-locking, its validity requires sufficiently slow medium dynamics. However, in QCLs with fast gain dynamics, and pronounced coherence effects such as RNGH instabilities, the conventional Master equation does not include coherent effects. Furthermore, the Haus Master equation only applies for amplitude-modulated combs, whilst free-running QCLs combs are frequency modulated, predominantly governed by the phase dynamic. However, the Haus Master equation has recently been developed into the coherent Master equation [44] and the Reduced Master equation [39]. By considering the nature of the fast gain dynamics, the coherent Master equation is more suitable for modeling QCLs. The reduced Master equation can be used to reproduce the behavior of frequency modulation combs in QCLs and study the roles of SHB, FWM and GVD on the pulse formation in QCLs.

## 3. RF Injection Locking of QCLs

Injection locking was originally used to transfer the spectral purity and stability of a master laser to a slave laser [48,49]. Typically, the master laser is a low-power spectrally pure laser, but the slave laser is a high-power and spectrally broad laser. When an optical seed from the master laser is injected into the cavity of the slave one, the slave laser can inherit the spectral and noise properties of the master laser. Simultaneously, the slave laser is able to maintain high power outcoupling. This technique shows great advantages for the amplification and stabilization of a single-mode laser. However, for broadband QCL it is not practical to realize the optical injection locking under the master oscillator power amplifier (MOPA) architecture as it requires locking many longitudinal modes together. Therefore, electrical injection locking has been developed to achieve this goal through locking the free spectral range (FSR) of the QCL to a microwave signal that is resonant with its round-trip frequency, instead of optically locking hundreds of longitudinal modes of the QCL, individually. Indeed, this approach has enabled active and hybrid mode-locking of inter-band semiconductor lasers [50,51,52]. Here, we emphasize this technique from theoretical and experimental aspects as it is the underpinning approach for ultrafast pulse generation and active mode-locking of QCLs.

When injection locking is presented, the beatnote of a laser has to be mentioned. In a QCL system, the beatnote is a series of discrete RF signals arising from the electrical beating of any two Fabry-Perot modes (*f_n+I_* − *f_n_*). For a 3-mm-long QCL cavity, the beatnote frequency is close to 13 GHz, as well as its harmonics at 26 GHz, 39 GHz… The fundamental beatnote is the most important parameter for active mode-locking, which can be expressed as the sum of the frequency difference between any two adjacent longitudinal modes as given in Equation (5):

(5) $$S\;(f)=\sum S_{i}\;\left(f_{n+1}-f_{n}\right)$$

The full width at half maximum (FWHM) of the beatnote signal is dominated by the frequency jittering of QCL modes. Similar to optical injection locking, by injecting an RF signal (*f*_RF_) that is resonant with the FSR or beatnote (*f*_beatnote_) into the QCL system, the spectral purity and stability of the low-noise external RF signal can be transferred to the QCL. From the complex amplitude evolution of the RF field in the system, the injection locking can be described using the following equation [53]:

(6) $$\frac{d\varphi }{dt}=\omega_{RF}-\Delta \omega -\omega_{L}sin\varphi$$

The injection-locking theory developed by *Adler* to describe the behavior of coupled nonlinear electronic oscillators [53]. However, it is ubiquitous in physical systems involving frequency locking between several oscillators such as lasers, mechanical oscillators, gyroscopes, etc. In Equation (6), *φ* represents the phase difference between the RF signal and the QCL internal electrical beating signal, ω_RF_ is the angular frequency of the RF signal, Δω is the angular frequency of the beatnote, and ω_L_ is the locking range, which can be further given in the following equation [54]:

(7) $$\omega_{L}=\frac{2\omega_{0}}{Q}\sqrt{\frac{P_{inj}}{P_{0}}}$$

In Equation (7), ω_0_ is the free-running oscillation angular frequency, Q is the oscillator q-factor, *P_inj_* is the injected power of RF source and *P*_0_ is the optical power within the laser cavity. When the condition |ω_RF_ − Δω| < ω_L_ is satisfied, Equation (6) has a steady-state solution: sin*φ* = (ω_RF_ − Δω)/ω_L_. In this case, the beatnote is locked to the injected RF signal and changes with it. When the condition |ω_RF_ − Δω| > ω_L_ is satisfied, Equation (6) will fall out of the locking range and the beatnote will no long be equal to the external modulation frequency. For mode-locking a QCL, the injected RF frequency and power has to satisfy the locking conditions given above.

Direct RF modulation was firstly introduced to THz QCL community in 2007 by Barbieri et al. [55]. They modulated the bias current that was injected into a THz QCL and observed the appearance of sideband modes in the emission spectrum, with a spacing that could be continuously tuned up to 13 GHz. The most important phenomena observed in the experiment was that when the modulation frequency approached the round-trip frequency of photons circulating in the resonant cavity, the number of QCL sidebands was considerably increased. This phenomenon, already observed in traditional lasers, was confirmed in QCL for the first time and was also in agreement with the above injection-locking theory. According to the Fourier transform, the broadened spectrum can potentially transfer to short pulses in time domain.

Thereafter, the injection locking of THz QCLs was studied in detail in the same group [54]. They investigated the longitudinal mode behavior of QCLs under different external modulation conditions. The first one was to fix the modulation frequency and change the RF modulation power. The second one was to fix the modulation power and change the modulation frequency. In both cases, a clear frequency “pulling effect” was observed as given in Ref [54]. They found a square-root dependence of the locking range with RF-power in agreement with classical injection-locking theory, as given in Equation (6). This THz QCL showed a locking range above 200 MHz, also in agreement with the theory described by Equation (7).

**Figure 2:**
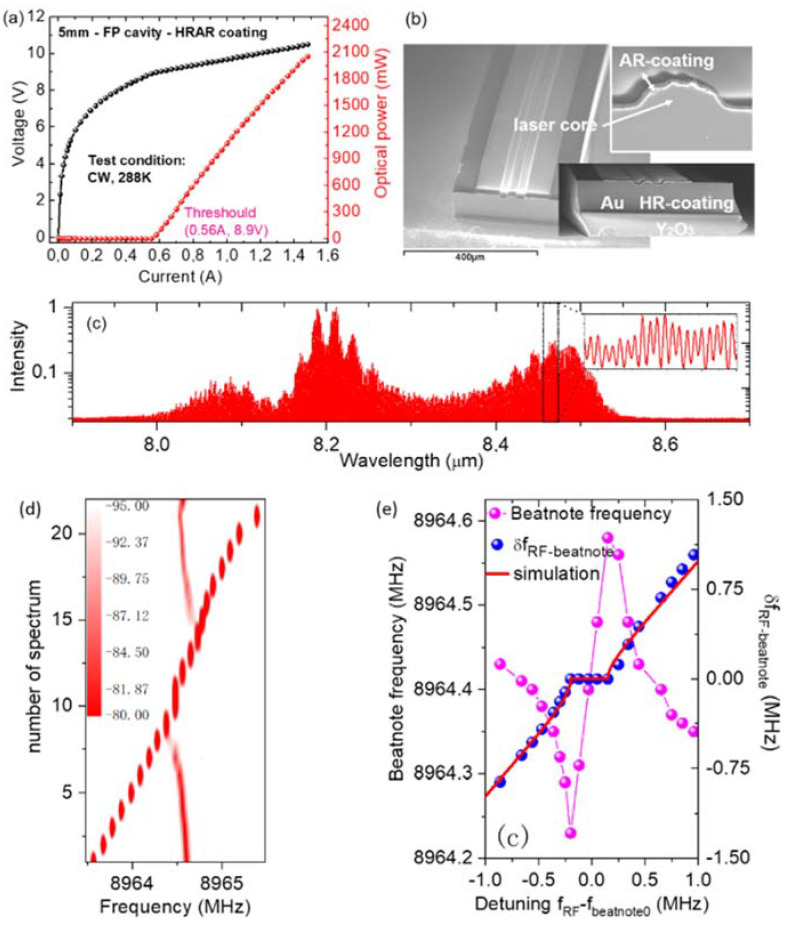
(**a**) The light-current-voltage (LIV) curves of a quantum cascade laser operating at room temperature in continuous wave. (**b**) The scanning electron microscope (SEM) image of the quantum cascade laser. (**c**) The spectrum of the laser. (**d**) The evolution of the beatnote (continuous branch) of the QCL as a function of the injected RF frequency (discrete branch) (each injected RF frequency can be found in the x axis). (**e**) Magenta: the beatnote frequency as a function of the detuning *δ* between RF frequency *f*_RF_ and the beatnote without RF injection Δ*f*_0_. Blue: the frequency difference *δf* RF−beatnote between RF and beatnote as a function of detuning *δ*. (Figure (**d**,**e**) from Ref [57], reprint with the permission of Optica publishing group).

Then, injection locking and harmonic injection locking were also demonstrated in mid-infrared QCL through direct microwave modulation [56,57]. As shown in the light-current-voltage (LIV) curve and the spectrum in Figure 2a,c, the QCL with a broadband emission spectrum spanning 8.0–8.6 μm is capable of delivering high optical power of over 2 W from a single facet in CW operation at approximately room temperature. Figure 2b also gives the scanning electron microscope (SEM) image of the high-power long-wave infrared QCL. Figure 2d shows the evolution of the beatnote (continuous branch) of the QCL as a function of the injected RF frequency (discrete branch). Figure 2e shows the beatnote frequency (magenta) as a function of the detuning *δ* between RF frequency *f*_RF_ and the beatnote without RF injection Δ*f*_0_. The blue shows the frequency difference *δf*_RF−beatnote_ between RF and beatnote as a function of detuning *δ*. From these two figures, we can clearly see that the beatnote frequency is locked to the injected RF frequency. However, the locking range is less than 1 MHz due to much higher intra-cavity power compared with the result in Ref [56]. This experiment was also in agreement with the injection-locking theory described by Equations (5)–(7).

## 4. Mode-Locked THz QCLs

The class of laser is the dominating factor regarding its transient behavior that determines how it generates short pulses. For QCL, the photon lifetime (τ_cav_) in laser cavity is in the order of magnitude 100–200 ps, while the lifetime of electrons (τ) on excited energy levels is in the order of a few picoseconds. This condition (τ_cav_ >> τ) gives an exponential growth transient behavior in the switched-on dynamic regime of QCL. Hence, QCLs are class-A lasers, which theoretically presents a difficulty for ultrafast pulse generation through the Q-switch technique. Mode-locking is, therefore, the only possible choice to generate ultrafast light pulses from QCLs.

**Figure 3:**
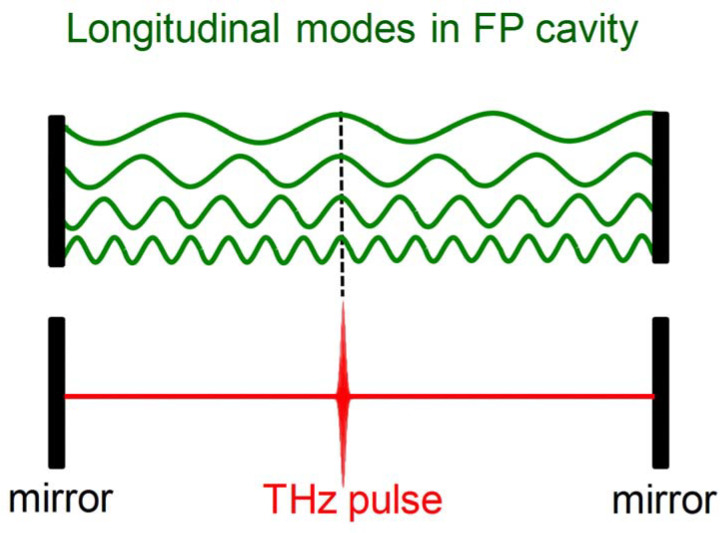
Schematic diagram of mode-locking in time or space domain in a Fabry-Pérot cavity.

Mode-locking is a widely used technique to generate ultra-short and intense pulses from lasers. Generally, when a laser is in operation, there is more than one resonant frequency that can be amplified and propagate in its cavity, as schematically illustrated in Figure 3 (green waves). These frequencies are called longitudinal modes of a laser, which are determined by the cavity length and the active medium of the laser. If all the modes are in phase and the mode spacing between these modes is identical, the electric field of all these modes will interfere constructively. This will result in an ultra-short and intense pulse (red in Figure 3 below) in the laser cavity. It will propagate back and forth within the cavity and then be partially coupled out from the cavity mirrors at every round-trip time. Temporally, a train of pulses separated by the laser cavity round-trip time will be obtained. This is the so-called mode-locking*:* to put all the longitudinal modes in phase (i.e., equal mode spacing Δω and time-independent phase φ) is the core technology for mode-locking.

If we suppose that the electric field of one longitudinal mode, for example the *m*^th^ one, is $E_{m}\left(t\right)=A_{m}e^{2\pi i\left(f_{m}^{\prime }t+\phi_{m}\right)}+c.c$. Adding the electric fields of all these resonant modes together will give us the laser emission in the time domain:

(8) $$\begin{array}{c} E(t)=\sum_{1}^{m}E_{m}(t)=\sum_{1}^{m}A_{m}e^{2\pi i(f_{m}^{\prime }t+\phi_{m})}+c.c\\ =\sum_{1}^{m}A_{m}e^{i\left[2\pi (f_{0}+m\cdot \delta f+\Delta f_{m})t+\phi_{m}\right]}+c.c\\ =\sum_{1}^{m}A_{m}e^{i\left[2\pi (f_{0}+m\cdot \delta f)t+\phi_{m}+2\pi \cdot \Delta f_{m}\cdot t\right]}+c.c\\ =\sum_{1}^{m}A_{m}e^{i\left[2\pi (f_{0}+m\cdot \delta f)t+\Phi_{m}(t)\right]}+c.c \end{array}$$

where *A_m_*, $f_{m}$ and $\phi_{m}$ are, respectively, the amplitude, frequency, and time-independent phase of the *m*^th^ mode. δω and $\Delta \omega_{m}$ are, respectively, the cold cavity mode spacing and the frequency-dependent mode shifting induced by hot cavity.$\;\Phi_{m}=2\pi \Delta f_{m}t+\phi_{m}$ is the time-dependent phase of the *m*^th^ mode and $\phi_{m}$ is the time-independent phase of the *m*^th^ mode.

**Figure 4:**
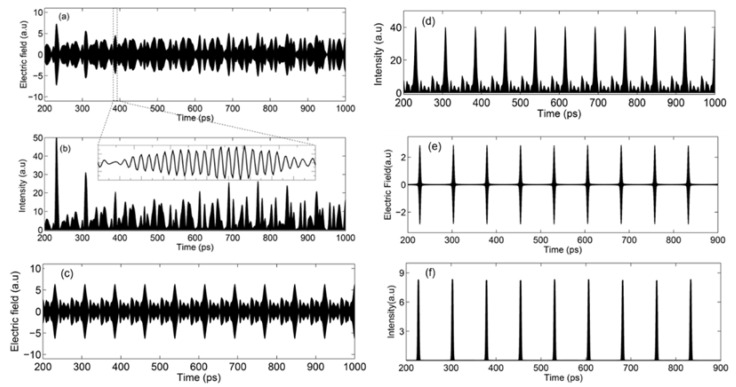
(**a**) Calculated electric field of laser emission from 200 ps to 1000 ps with a femtosecond time resolution with a time-dependent phase and φ_m_ = 0. (**b**) Intensity of laser emission corresponding to (**a**). (**c**,**d**) Calculated lasers emission with Δ*f*_m_ = 0 but phase ϕ_m_ of each mode is different: (**c**) Electric field from 200 ps to 1000 ps and (**d**) Intensity corresponding to (**c**). (**e**) Electric field of a mode-locked laser emission as a function of time. (**f**) Intensity corresponds to the electric field above.

We now consider how these parameters affect the temporal behavior of the electric field. The time-dependent phase varies between modes and is always changing with time. This will bring a ‘random (unfixed) phase-relation’ between the modes at any time and will result in continuous (non-periodic) wave emission in time, as shown in Figure 4a,b, which is calculated from Equation (8). We can see that the emission is not periodic, due to non-equal mode spacing bringing a time-dependent floating phase. This is why even a broadband laser does not give us stable pulse emission under free-running conditions. Now, if Δ*f*_m_ = 0 (the spacing between these modes is constant), but *ϕ_m_* varies for all modes, laser emission will become periodic, with pulses being observed, but the pulse shape will be heavily deformed, as shown in Figure 4c,d. If a laser is mode-locked, Δ*f*_m_ = 0 and *ϕ_m_* = 0. Adding all these modes together, the emissions of a laser in time domain will become periodic Gaussian pulses, as shown in Figure 4e,f.

As discussed above, the key mission of mode-locking is to remove or fix the time dependent phase term and make the mode spacing and phase of a laser to be identical. Generally, when a laser is mode-locked and periodic pulses are generated, δf and $\phi_{m}$ will be automatically fixed due to the ‘‘phase-matched’’ modulation imposed on these modes.

How can we fix the free spectral range *δf* of a laser and keep the modes in phase? There are many ways that can be used to achieve this, including active mode-locking, passive mode-locking and hybrid mode-locking. Each type of mode-locking can be also realized by many different detailed techniques, such as direct current modulation [58], acoustic-optic modulation [59], saturable absorption [60], and nonlinear Kerr effect [61], etc.

**Figure 5:**
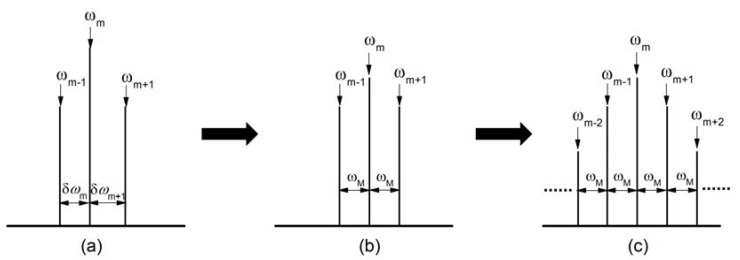
Schematic diagram of active mode-locking in frequency domain. (**a**) Spectrum of free-running emission. (**b**) Modulation is applied on a laser. (**c**) Laser is mode-locked.

Here we present active mode-locking as it is the most adapted for pulse generation from QCLs. Generally, we employ an electrical modulation *ω_M_*, which is monochromatic and very close to the mode spacing δ*ω*, to modulate a laser directly (i.e., modulation at the round trip of the cavity). Firstly, let us consider the modulation effect on the frequency *ω_m_*, as is illustrated in Figure 5a. Before modulation is applied, the free-running emission mode spacings are not identical δ*ω_m_* ≠ δ*ω_m_*_+1_. When modulation is applied, the central frequency *ω_m_* will transfer a part of its energies to its modulated sidebands (*ω_m_* + *ω_M_*, *ω_m_* − *ω_M_*) and will be close in frequency to the two free-running modes (*ω_m_*_−1_, *ω_m_*_+1_) of the cavity. If the modulation power is strong enough, it will force the free-running frequencies (*ω_m_*_−1_, *ω_m_*_+1_) to move towards the sidebands’ frequencies positions at (*ω_m_* + *ω_M_*, *ω_m_* − *ω_M_*) until they totally overlap *ω_m_*_−1_ = *ω_m_* − *ω_M_*, *ω_m_*_+1_ = *ω_m_* + *ω_M_*. Finally, the mode spacing will be locked to the modulation frequency δ*ω_m_ =* δ*ω_m_*_+1_ = *ω_M_*, as presented in Figure 5b.

Above we have analyzed the modulation effect only on the central frequency *ω_m_*. We can analyze the other frequencies in the same way: *ω_m_*_−1_ to obtain *δω_m_*_−1_ *=* δ*ω_m_* = *ω_M_*, *ω_m_*_−2_ to get *δω_m_*_−2_ *= δω_m_*_−1_ = *ω_M_*, *ω_m_*_−3_ to obtain *δω_m_*_−3_ *= δω_m_*_−2_ = *ω_M_*…. At the end, we have *δω_m_*_−2_ *= δω_m_*_−1_ = *δω_m_ = δω_m_*_+1_ *= δω_m_*_+2_ *= … = ω_M_*, i.e., the mode spacing over the whole spectrum of a laser emission will be fixed to the modulation frequency, as shown in Figure 5c. Simultaneously, the time-independent phase *ϕ_m_* will also be identical among all the modes, due to the synchronization of the modes through the active modulation. As mentioned above in Figure 4, once the mode spacing is locked and the phases are identical, the laser emission in the time domain will be pulsed and active mode-locking will be realized.

Unlike traditional semiconductor lasers, the QCL transition takes place between two inter-sub-band energy levels originating from nanoscale confinement of electrons in quantum wells. As mention above, this leads to fast gain recovery time, orders of magnitude shorter than in interband lasers [19,20,21,23], and a time considerably shorter than the photon round-trip cavity time. This is believed to prevent these devices from being mode-locked (multiple pulses are generated within the QCL cavity) and, thus, unable to generate short pulses using passive approaches.

However, it has been shown recently that these devices can be actively mode-locked, where the QCL is modulated at microwave frequencies, to generate a train of picosecond pulses [22,62]. The key to these demonstrations has been the development of new ultrafast techniques for the THz range. In the first case, detection of the emitted pulse train has been made possible by phase-locking the QCL repetition rate and carrier frequency to a high order harmonic of the repetition rate of a mode-locked femtosecond laser. This technique permits coherent detection of the THz electric field, and allows the control of the carrier-envelope phase shift of the QCL. Its disadvantage is that it undersamples the electric field of the pulse train of lasers in the time domain.

**Figure 6:**
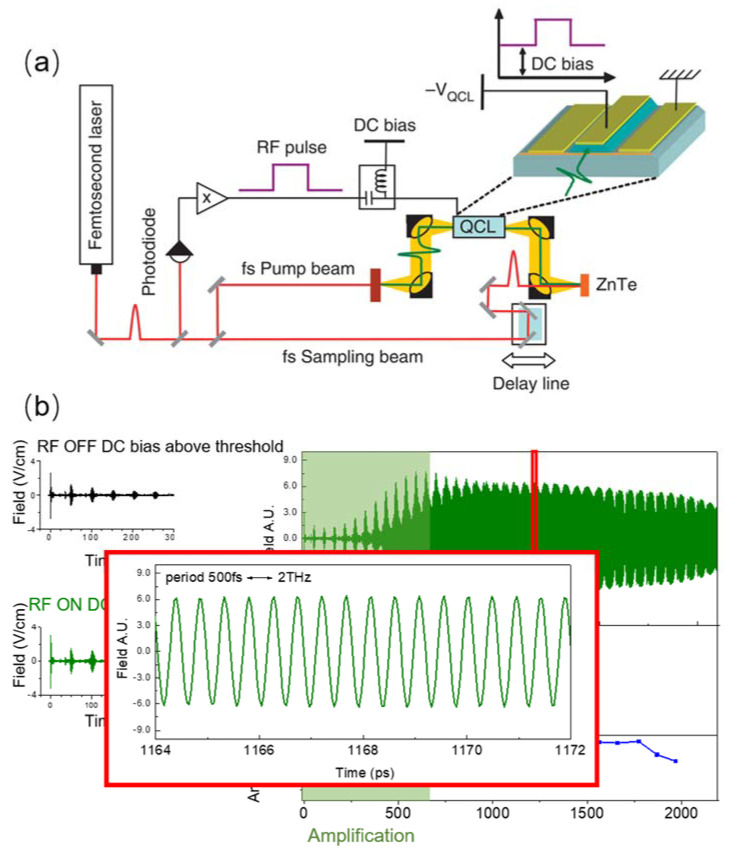
(**a**) Schematic of the experimental setup. RF voltage pulses are generated from a fast photodiode illuminated by the femtosecond laser beam. The RF pulses are amplified by a power RF amplifier. A bias tee adds a DC offset to the RF pulses. THz pulses are generated by illuminating a biased interdigitated antenna with a femtosecond pump beam. The THz pulses are coupled into a facet of the QCL with parabolic mirrors. The QCL output field from the other facet is measured using electro-optic sampling in a ZnTe crystal with a femtosecond sampling. (**b**) The experimental result: full electric field resolved emission of a THz QCL working at 2 THz. (Figure (**a**) from Ref [63], licensed under a Creative Commons Public (CCPL) license).

An alternative ultrafast detection technique called the “injection seeding technique” has also been developed [63]. This technique has the full capability to measure all the information of QCL emission in time domain, including phase, amplitude, intensity, spectrum, and full electric field, as shown in Figure 6. This provides the possibility to observe directly pulse-train generation and has paved the way for QCL mode-locking demonstration directly in time domain.

**Figure 7:**
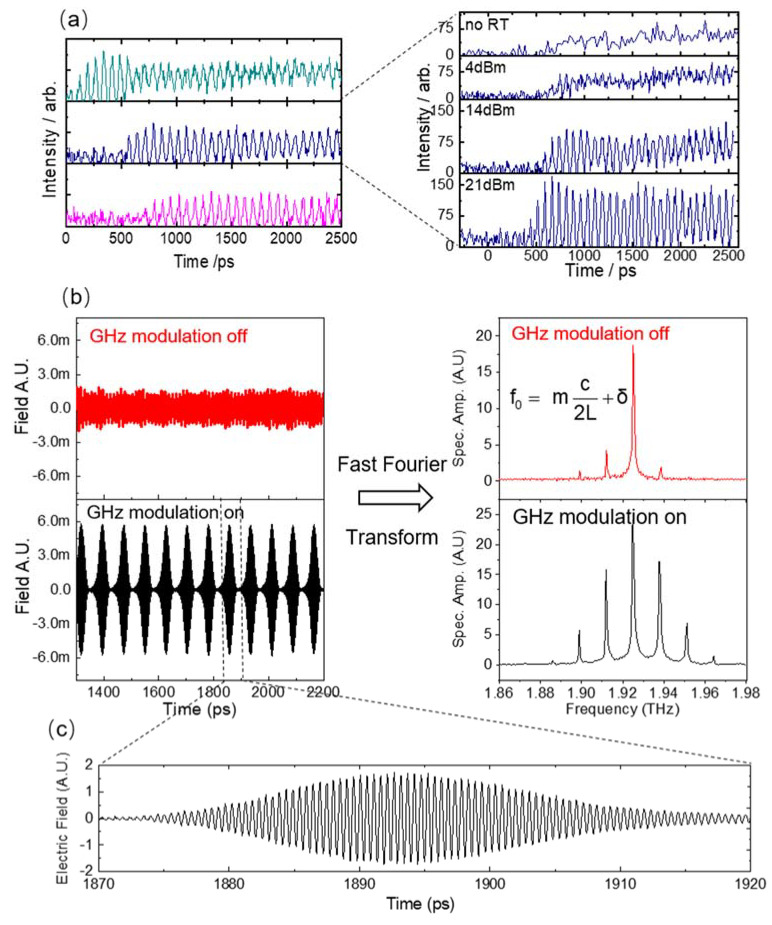
(**a**) Sampled THz intensity from the QCL. Left: at different currents. Right: a different modulation power. The detailed explanation on different color can be found in Ref [32] (**b**) The electric field (left) and its corresponding spectra (right) of QCL emission with and without round-trip modulation, respectively. (**c**) The zoom in of one THz pulse of QCL emission. (Figure (**a**) from Ref [32], reprint with the permission of AIP Publishing).

Immediately after the development of this injection seeding technique, mode-locking of THz QCLs was realized and demonstrated in time domain [32,33,34,64]. A series of important work was published on this research topic, showing that THz QCLs could be mode-locked for short pulse generation. Figure 7a shows the THz intensity emitted by an actively mode-locked QCLs over picosecond time scales (without a seed). Both the initiation of mode-locked pulses and the steady-state regime were examined. For bias conditions well above threshold, a sinusoidal modulation of the emission was achieved; however, when the QCL was biased around threshold and the round-trip modulation was strong, Gaussian-shaped transform limited mode-locked pulses with a full width at half maximum (FWHM) of 19 ps were observed. Figure 7b shows the electric field (left) and its corresponding spectra (right) of QCL emission with and without round-trip modulation, respectively, in the time domain using the injection seeding technique. The method relied on synchronizing the mode-locked pulses to a reference laser and was applied to 15-ps pulses generated by a 2-THz QCL. The pulses from the actively mode-locked laser were completely characterized in field and in time with a sub-ps resolution, allowing us to determine the amplitude and phase of each cavity mode. Figure 7c shows the zoom in of a light pulse from the mode-locked QCL. We can clearly resolve the oscillation of the electric field of the laser emission.

**Figure 8:**
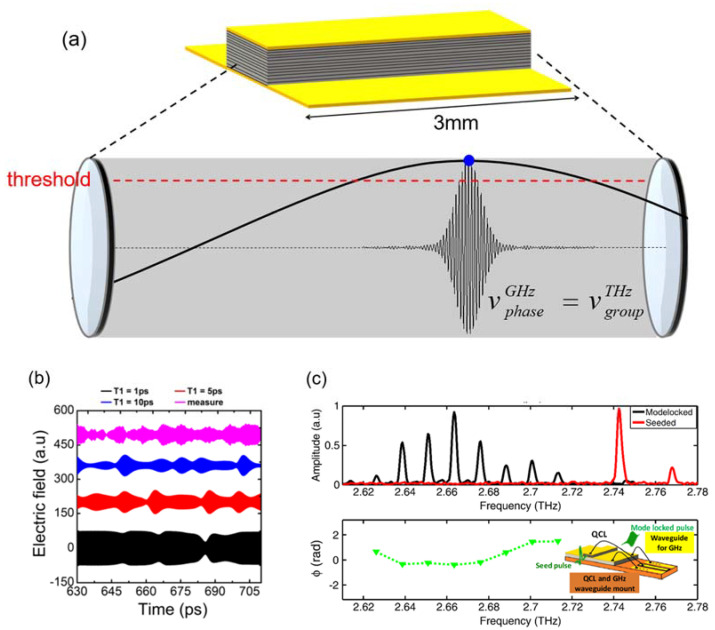
(**a**) The schematic of mode-locking mechanism of QCLs. (**b**) The Maxwell–Bloch simulations of the gain recovery time: Calculated time-domain profiles of QCL output emission over one round-trip period for gain recovery times of 1 ps (black), 5 ps (red) and 10 ps (blue curve). The magenta curve corresponds to experimental data. (**c**) Top: the spectra of a seeded (red) and a mode-locked (black) QCL, bottom: the phases of the eight mode-locked longitudinal modes (green triangles). Inset: QCL schematic showing the device integrated with a microwave waveguide for active mode-locking and illustrating the input seed pulse and the seeded/mode-locked output. (Figures from Ref [23], reprint with the permission of Optica publishing group).

Since then, mode-locked THz QCLs have been experimentally demonstrated using different detection approaches as discussed above. However, the exact mechanism of mode-locking in QCLs is still unknown, which strongly limits new avenues to be explored to generate shorter and more intense laser pulses. Over a series of samples and measurements by researchers [23], it has been found that, contrary to a long-standing belief that the QCL gain dynamics are the limiting factor, the key mechanism is in fact a nonlinear interaction between the pulse generated and the applied electrical modulation [23], as shown in Figure 8a. This is important information and has permitted new avenues to be explored to generate shorter and intense pulses.

Figure 8b shows the Maxwell-Bloch simulations of the gain recovery time (T_1_). It was calculated using Maxwell-Bloch finite-difference time-domain simulations in a two-level system [23]. The procedure is detailed in depth in Ref. [31]. Here, a dephasing time about 0.6 ps from the full-width at half-maximum of the gain and a total waveguide loss of 12 cm^−1^ from the first pass gain measurements of the longitudinal optical (LO) phonon-depopulation-based QCL were used. A time data with a gain recovery time of ~5 ps showed the best ‘fit’ with the data. The ultrafast gain recovery time measured here, which did not limit pulse generation, could be used as an advantage to generate more intense and shorter pulses if short intense electrical pulses could be used to switch on the QCL gain. For example, a Gaussian or Lorentzian profile could be used. Although difficult to generate electronically, optically generated electrical pulses using ultrafast lasers combined with ultrafast materials are feasible and these could then be used to switch the QCL on sub-picosecond time scales. Further techniques that could circumvent the current limitations would be the application of greater microwave power for higher pulse energies and the application of hybrid mode-locking techniques to shorten the pulses to sub-10 ps values. Figure 8c top shows the spectra of a seeded (red) and a mode-locked (black) QCL; bottom shows the phases of the eight mode-locked longitudinal modes (green triangles).

## 5. Pulse Shortening in Mode-Locked THz QCLs

As of 2013, active mode-locked THz QCLs have been demonstrated through different measures in different groups. However, the pulse width of mode-locked QCLs is quite large, falling between 10 and 20 ps. Researchers have attempted many different approaches, including using broad-bandwidth QCLs, designing different geometry structures, adopting hybrid mode-locking techniques, etc., to compress the pulse width below 10 ps but without any success despite many active research efforts [54,56,57,65].

In 2016, the research group at TU Wien Vienna showed that a single THz pulses as short as 2.5 ps could be generated from a QCL [66]. However, this was not a train of pulses, with subsequent pulses broadening as the QCL was not actively mode-locked.

**Figure 9:**
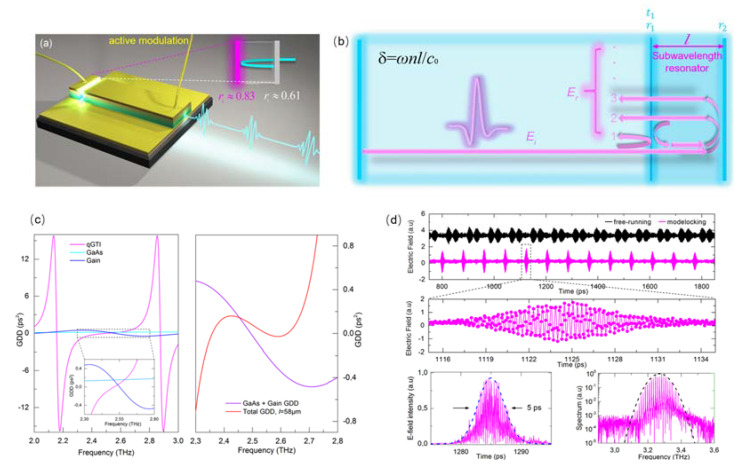
(**a**) The schematic of short-pulse generation based on on-chip dispersion compensation. (**b**) The schematic of dispersion compensation based on Gires–Tournois Interferometer. (**c**) Group Delay Simulations of the gain, material and GTI. Left: The individual GDD contributions of the GTI, GaAs and the QCL gain. Right: The total GDD for a 58μm (red) length GTIs. The contribution of the gain and material GDD is also shown for comparison. (Figure (**d**) from Ref [26], licensed under a Creative Commons Attribution (CC BY) license).

To realize a mode-locked pulse train, a monolithic on-chip dispersion compensation scheme to shorten the THz pulses of mode-locked QCLs was proposed [26]. This was based on the realization of a small coupled cavity resonator that acted as an ‘off resonance’ Gires–Tournois interferometer (GTI), permitting large THz spectral bandwidths to be compensated, as shown in Figure 9. In this work, the THz pulses of mode-locked QCLs was considerably shortened from 16 ps to 4 ps. This permitted the compression of THz pulses of mode-locked QCLs beyond the 10 ps barrier that had stood for several years. This result marks an important milestone in exploring ultrafast light-pulse generation from mode-locked QCLs. The novel application of a GTI also opens up a direct route to sub-picosecond and single cycle pulses in the THz range from a compact semiconductor source.

## 6. Pulse Generation in Mid-Infrared QCLs

The development of mid-infrared QCLs is far ahead of THz QCLs [3,4,6,7,8,67], but its mode-locking is lagging behind the THz QLCs due to an even shorter gain recovery time (~1 ps). Following the mode-locking of THz QCLs, generating ultrashort pulses from mid-infrared QCLs has also undergone considerable improvement in the past a few years. The first experimental demonstration was realized in 2009 by Capasso’s group at Harvard [62] and the theoretical demonstration of active mode-locking of such QCLs was reported in 2015 by Belyanin’s group in Texas [25]. They investigated the dynamics of actively modulated mid-infrared QCLs using space- and time-domain simulations of coupled density matrix and Maxwell equations, with resonant tunneling current taken into account. They showed that it was possible to achieve active mode-locking and stable generation of picosecond pulses in QCLs by bias modulation of a short section of a monolithic Fabry–Pérot cavity.

**Figure 10:**
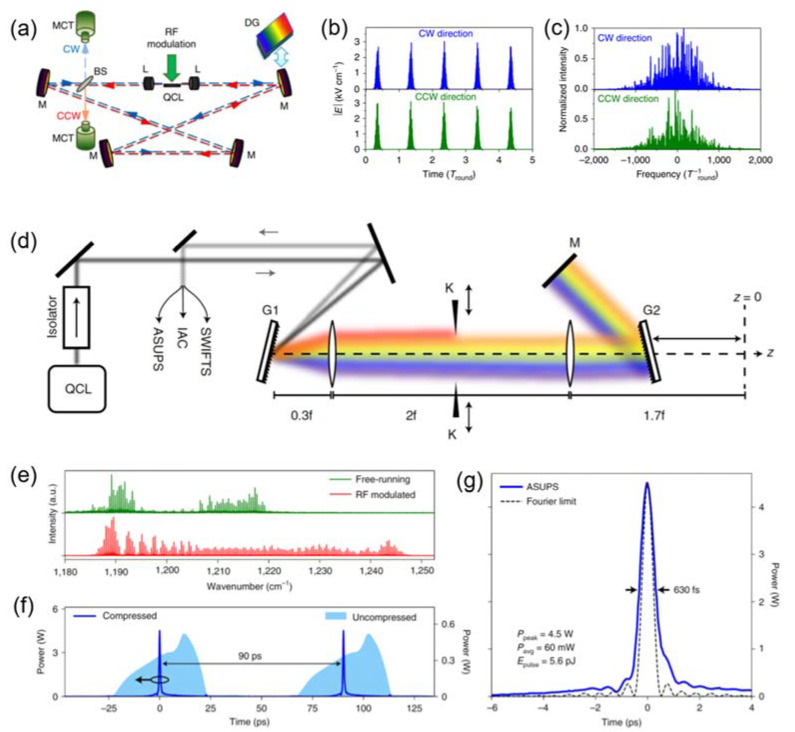
(**a**) Optical set-up of free-space external ring cavity quantum cascade laser. BS: beam splitter; CCW: counter-clockwise direction; CW: clockwise direction; DG: diffraction grating; L: aspheric lenses; M: mirrors; MCT: detector. (**b**) Absolute values of the electric fields and (**c**) their spectra for a sinusoidal modulation of bias with the modulation period equal to 1.01 T_round_. (**d**) The experimental setup for pulse compressing using diffraction grating compressor. (**e**) Free-running and RF-modulated optical spectra of mid-infrared QCL emission. (**f**) The intensity profile of the QCL before and after pulse compression. (**g**) The shortest pulse obtained with a pulse length of 630 fs with a peak power of 4.5 W. (Figure (**a**–**c**) from Ref [24], licensed under a Creative Commons Attribution (CC BY) license. Figure (**d**–**g**) from Ref [27], licensed under a Creative Commons Attribution (CC BY) license).

In the same year, active mode-locking of mid-infrared QCLs at a wavelength of 5 μm was experimentally demonstrated in a free-space external ring cavity QCL, as shown in Figure 10a [24]. The laser operated at room temperature and stayed in mode-locking state over the full dynamic range of injection currents. Figure 10b,c shows the estimated pulse width and corresponding spectra using a four-subband model for the QCL active region, which ranges between 10 ps and 45 ps depending on the cavity length. In the paper, the theoretical modeling showed that one could achieve much shorter pulses and broader phase-locked frequency combs by modulating the pumping with shorter and sharper pulses instead of the sinusoidal modulation. This finding is completely in agreement with the experimental observation in Ref. [23].

Recently, Faist’s group at ETH Zurich also demonstrated an approach capable of producing near-transform-limited sub-picosecond pulses (630 femtosecond) with several watts of peak power at a wavelength of around 8μm using a diffraction grating compressor, as shown in Figure 10d [27]. Starting from a frequency-modulated phase-locked state, ultrashort high-peak-power pulses were generated via spectral filtering, gain modulation-induced spectral broadening, and external-pulse compression. They investigated the pulse width of QCLs emission using a novel asynchronous sampling method, coherent beatnote interferometry, and interferometric autocorrelation. Figure 10e shows the free-running and round-trip-modulated optical spectra, respectively. It can be clearly observed that a considerable increase in spectral bandwidth has been achieved in the latter case. Such a temporal modulation brings a strong overall amplitude modulation, accomplished with the decrease of emitted average power due to increased gain saturation, as shown in Figure 10f. This is another milestone in ultrafast pulse generation from QCLs following the 4 ps THz pulse generation from mode-locked THz QCLs. These achievements presented above are also listed in Table 1 given below:

## 7. Conclusions and Perspectives

To conclude, pulse generation through mode-locking of QCLs has undergone considerable development in the past decade. Owing to the fast dynamics, QCLs were thought to be very difficult to mode-lock. Through active mode-locking and pulse compression, an ultrashort pulse train as short as 4 ps in THz and 0.6 ps in mid-infrared regime has been realized from mode-locked QCLs. These results push QCLs to a new milestone, enabling a range of applications in fundamental research, high-tech industry and defense technology, particularly in mid-infrared and THz nonlinear optics where high pulse energies are typically required. With further development of this technology, many new QCL-based applications will emerge in the near future, potentially replacing or being complementary to OPA technologies.

## Figures and Tables

**Table 1:** Achievements on pulse generation from QCLs.

	Pulse Width	Wavelength/ Frequency	Operation Temperature	Method	Peak Power
2009 Ref. [62]	3 ps	6.3 μm	77 K	active modulation	0.5 pJ
2011 Ref. [22]	10 ps	2.5 THz	20 K	coherent sampling	×
2012 Refs. [32,33,34]	10–20 ps	2 THz	10 K	active modulation	×
2016 Ref. [24]	10–45 ps	5.25 μm	300 K	external cavity	12 mW
2017 Ref. [26]	4 ps	2.2–2.8 THz	20 K	dispersion compensation	×
2021 Ref. [27]	0.63 ps	8 μm	300 K	external pulse compression	4.5 W
